# Clinical analysis of Bornavirus Encephalitis cases demonstrates a small time window for Etiological Diagnostics and treatment attempts, a large case series from Germany 1996–2022

**DOI:** 10.1007/s15010-024-02337-3

**Published:** 2024-07-19

**Authors:** Kirsten Pörtner, Hendrik Wilking, Christina Frank, Klaus Stark, Silke Wunderlich, Dennis Tappe

**Affiliations:** 1 Department of Infectious Disease Epidemiology, Robert Koch Institute, Seestr. 10, 13353 Berlin, Germany; 2 School of Medicine, Department of Neurology, Technical University of Munich, Munich, Germany; 3 Reference Laboratory for Bornaviruses, Bernhard Nocht Institute for Tropical Medicine, Hamburg, Germany

**Keywords:** Bornavirus, BoDV-1, VSBV-1, Encephalitis

## Abstract

**Purpose:**

The emerging zoonotic Borna disease virus 1 (BoDV-1) and the variegated squirrel bornavirus 1 (VSBV-1) cause severe and fatal human encephalitis in Germany. We conducted the first systematic clinical analysis of acute, molecularly confirmed fatal bornavirus encephalitis cases comprising 21 BoDV-1 and four VSBV-1 patients to identify options for better diagnosis and timely treatment.

**Methods:**

Analyses were based on medical records and, for BoDV-1, on additional medical interviews with patients’ relatives.

**Results:**

Disease onset was unspecific, often with fever and headache, inconsistently mixed with early fluctuating neurological symptoms, all rapidly leading to severe encephalopathy and progressive vigilance decline. Very shortly after seeking the first medical advice (median time interval 2 and 0 days for BoDV-1 and VSBV-1, respectively), all except one patient were hospitalised upon manifest neurological symptoms (median 10 and 16 days respectively after general symptom onset). Neurological symptoms varied, always progressing to coma and death. BoDV-1 and VSBV-1 patients required ventilation a median of three and five days, and died a median of 32 and 72 days, after hospitalisation. Death occurred mostly after supportive treatment cessation at different points in time based on poor prognosis. Disease duration therefore showed a wide, incomparable range.

**Conclusion:**

The extremely rapid progression is the most obvious clinical characteristic of bornavirus encephalitis and the timeframe for diagnosis and targeted therapy is very short. Therefore, our results demand an early clinical suspicion based on symptomatology, epidemiology, imaging, and laboratory findings, followed by prompt virological testing as a prerequisite for any potentially effective treatment.

## Introduction

The Borna disease virus 1 (BoDV-1; species *Orthobornavirus bornaense*) and the variegated squirrel bornavirus 1 (VSBV-1; species *Orthobornavirus sciurini*), members of the *Bornaviridae* family, typically cause fatal encephalitis in humans. Human infections have only been detected in Germany, the first cases being reported in 2018 [1, 2] and 2015 [3], respectively. As of mid-2024, around 50 molecularly confirmed BoDV-1 cases are known, including retrospective diagnosis, all presenting with fulminant encephalitis. The Robert Koch Institute estimates 5–10 incident BoDV-1 cases per year. Only a total of five confirmed VSBV-1 encephalitis cases are known [4] - one with a chronic 12-year-long course [5], excluded from this study.

Active case finding and serological studies among populations at risk [5, 6] indicated a high clinical manifestation index of bornavirus infection, presenting as severe encephalitis. A single-centre study found BoDV-1 in archived brain specimens in seven out of nine cases with fatal encephalitis of previously unknown etiology [7]. Neither an effective therapy nor a vaccine is established. Favipiravir has shown in vitro effects [8], but in vivo data are lacking.

BoDV-1 is harboured at least by the shrew *Crocidura leucodon* [9], with endemic areas in parts of Germany and neighbouring countries. VSBV-1 was found in exotic squirrels in private holdings and zoos in Germany and other European countries; its geographic origin is still unknown [4, 10]. Infected shrews and squirrels harbour the respective viruses in their nervous system and in organs of excretion [11, 12]. Risk factors for either form of bornavirus encephalitis are hard to elucidate, encompassing so far.

- for BoDV-1 residence on the fringe of settlements in rural virus-endemic areas [13].
- for VSBV-1 contact to exotic *Sciurinae* or *Callosciurinae* squirrels [3, 5, 14].

The mode of transmission and the portal of entry in humans is unknown, with an olfactory route [15] from an indirect shrew-infested environmental source [13] discussed for BoDV-1, and possibly scratches and bites by exotic squirrels [3, 14] or also via the olfactory route [16] for VSBV-1. The incubation period comprises likely weeks to months [17].

Whereas data on the clinical picture and the course of disease in humans is limited and has been presented individually in single cases and small case series for BoDV-1 [1, 2, 5, 18–25] and VSBV-1 [3, 5, 14, 26] only, cerebrospinal fluid (CSF) changes [27, 28], magnetic resonance imaging (MRI) findings [16, 29], and neuropathology results indicative of immune-mediated pathogenesis [28, 30, 31] were systematically performed. Notably, CSF and MRI changes are characteristic but not specific and can be absent at baseline [16, 27–29, 32]. Early diagnosis therefore remains challenging, and is complicated by late seroconversion and low sensitivity of PCR in CSF, at least for BoDV-1 [22, 33]. Knowledge of the clinical presentation and the course of disease as well as differential diagnoses could help clinicians in establishing a rapid diagnosis and might allow for a targeted therapy.

Here, we report the first systematic analysis of the clinical disease course of bornavirus encephalitis in the so far largest case series available. Analyses were not only based on medical records, but for BoDV-1 also on structured medical interviews with patient relatives. Enabling an early diagnosis of bornavirus encephalitis by identifying critical points in time and clinical characteristics to initiate treatment attempts was the aim of this study.

## Patients and methods

*BoDV-1 Encephalitis.* We analyzed the clinical course of 21 molecularly confirmed acute BoDV-1 infections (12 female, 9 male), including single cases previously published with very limited individual clinical information based on medical records [1, 5, 7, 15, 18–22, 24, 29, 31] and clinically non-published cases. Median age was 47 (range 6–79) years at disease onset. The cases were geographically and/or temporally sporadic without evidence of common sources or person-to-person transmission; all cases lived very rurally in areas endemic for animal BoDV-1 disease. The interview methodology, detailed epidemiological data on all 21 cases, a map of the residencies within the endemic areas (based on animal data), and epidemiological risk factors were recently published elsewhere [13, 15]. As all patients were deceased, 1–2 household or close family members were approached as proxies by a clinician and agreed to detailed comprehensive interviews about the development and sequence of clinical symptoms. Dermatological, gastrointestinal, upper and lower respiratory, psychiatric and neurological signs and symptoms were asked, as well as general symptoms of a viral infection, such as fever, headache, fatigue, malaise, anorexia, myalgia and arthralgia. Symptom onset was defined as any new symptom reported by the interviewed relative(s)/household member(s) being in line with the onset of a local viral infection, including gastrointestinal and respiratory symptoms, as well as signs reflecting a systemic reaction (for example fever, fatigue, malaise), followed by new neurological symptoms up to 12 months before progressing to encephalitis.

Interview data was complemented by the respective medical records (available for 16/21, 77%) and/or compared to the respective publications of some of the individual cases [15, 18, 21, 29, 31]. Four known chronic BoDV-1 encephalitis cases (among them three without molecular confirmation due to the lack of brain samples and serological/epidemiological diagnosis only, Source: Robert Koch Institute) with an unusual disease duration of more than a year, all presenting with a fulminant clinical course and long-term sequelae, were not included in this study.

*VSBV-1 Encephalitis*. We investigated the clinical course of four molecularly confirmed acute cases (three males, one female). Median age was 62 (range 45–72) years at disease onset. Three cases formed the initial encephalitis cluster among inter-trading private exotic squirrel breeders leading to the discovery of VSBV-1 [3]. The fourth case pertains to a professional animal caretaker [14].

If prodromal (non-neurological) signs were absent, any new neurological symptom prior to progression to encephalitis was defined as disease onset in either form of bornavirus encephalitis. Neither BoDV-1 nor VSBV-1 infected patients had been on immunosuppression or had noteworthy pre-existing conditions.

Interviewees received written information about the study and the data protection concept and family members of BoDV-1 and VSBV-1 patients were asked to provide clearance for medical charts. Ethical clearance was obtained prior from the Medical Board of Hamburg (no. PV5616).

## Results

### Temporal distribution of cases and diagnosis

**Fig. 1:**
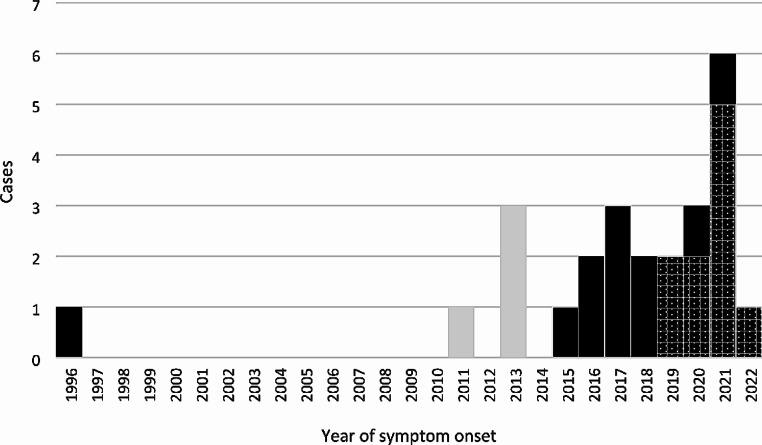
Temporal distribution of 21 interviewed, confirmed acute BoDV-1 (solid black and dotted black), and four VSBV-1 encephalitis cases (grey) and time of diagnosis. BoDV-1 cases with *intra vitam* diagnosis are symbolized by dots. Cases in solid black or grey were diagnosed retrospectively. Only in 2018 BoDV-1 became known as causal agent of human encephalitis [1, 2]. In 2015 VSBV-1 became known as causal agent of human encephalitis [3]

Figure 1 shows the temporal distribution of included BoDV-1 and VSBV-1 cases and the time of diagnosis. Diagnosis *post mortem* was mostly in cases incident prior to the discovery of the viruses in 2018 and 2015, respectively.

*BoDV-1 Encephalitis.* Diagnosis *intra vitam* was accomplished after 2018, the year of the discovery of the virus. A definite diagnosis in the 10 cases with *intra vitam* diagnosis (PCR in CSF or brain tissue) was established a median of 17 (IQR 9–24) days after hospitalisation, all cases already intubated.

*VSBV-1 Encephalitis.* In the four cases included here, VSBV-1 was diagnosed retrospectively *post mortem* in archived CSF or brain tissue by PCR.

### Initial signs and symptoms, and First Medical Consultation

*BoDV-1 Encephalitis.* Before developing encephalopathy or focal neurological signs, 14/21 patients experienced other, non-neurological, prodromal signs and symptoms. Prodromal symptomatology was unspecific, insidious, fluctuating and often only recognized by the interviewees retrospectively when explicitly queried. The time interval with prodromal symptoms before hospitalisation varied from one to 75 days maximum, and was in median 10 days. In the majority of patients prodromes included strong headaches, and fever, as well as other flu-like symptoms such as extreme fatigue, malaise, upper respiratory tract symptoms, and gastrointestinal symptoms. In seven patients, the first reported symptoms of disease were those of encephalopathy or focal neurological signs without any preceding known non-neurological symptoms (but with concurrent or developing non-neurological symptoms in the clinical course). The first medical advice was sought a median of seven days (IQR 3–10) after general symptom onset. The spectrum of early neurological symptoms (in patients with prodromes or without) was broad. Vigilance decline was the most predominant neurological finding (19/21, 90%), (including confusion, disorientation, memory disturbance, and psychomotor slowing), others included personality changes, dysarthria, aphasia, coordination difficulties, epileptic seizures, and syndrome of inappropriate antidiuretic hormone secretion (SIADH), and affected the central nervous system in all but one case: Only the family of the case deceased in 1996 [18] did not report any central but peripheral neurological difficulties (compatible to Guillain-Barré syndrome) before admission. Also, very strong (neurogenic) pain in the shoulder, the neck or the jaw were reported in two patients, leading to the extraction of the wisdom teeth in one patient. In the majority of all 21 cases, an exact day of symptom or disease onset could not be reported.

*VSBV-1 Encephalitis.* All four patients experienced non-neurological prodromal symptoms before developing encephalopathy or focal neurological deficits, including fever and/or shivers, fatigue, malaise, cough, sore throat, or abdominal discomfort. Early neurological symptoms followed and encompassed vigilance decline, drowsiness, ataxia or unsteady gait, paraesthesia or neurogenic pain, and myoclonus. In three cases, medical advice was sought because of deterioration of the general condition, especially when vigilance decline increased, and in one case because of incremental fever on top of upper respiratory tract symptoms, all as hospital outpatients/emergency department consultation. All patients had at least one flu-like symptom and one sign of neurological involvement before hospital admission. Due to incremental development of early signs and symptoms, an exact day of onset was not recorded.

Table 1 summarizes signs and symptoms of BoDV-1 and VSBV-1 cases before hospital admission.

**Table 1:** Signs and symptoms of BoDV-1 and VSBV-1 encephalitis cases and their frequency of occurrence according to household/family members and/or medical records *before* hospital admission

Signs and symptoms before hospital admission	Frequency in BoDV-1 encephalitis patients (*n* = 21) according to family members and medical records where available	Frequency in VSBV-1 encephalitis patients (*n* = 4) according to medical records
Fever/Shivers	14/21 (67%)	3/4 (75%)
Headache	13/21 (62%)	2/4 (50%)
Fatigue	13/21 (62%)	2/4 (50%)
Malaise	15/21 (71%)	1/4 (25%)
Abdominal Pain/Discomfort	2/21 (10%)	2/4 (50%)
Vomiting	10/21 (48%)	0/4 (0%)
Diarrhea	2/21 (10%)	0/4 (0%)
Cough	4/21 (19%)	1/4 (25%)
Rhinitis/sinusitis	1/21 (5%)	1/4 (25%)
Otitis	1/21 (5%)	0/4 (0%)
Sore Throat	1/21 (5%)	2/4 (50%)
Non-neurological prodromes preceding neurological signs and symptoms	14/21 (67%)	4/4 (100%)
*Neurological signs and symptoms*	21/21 (100%)	4/4 (100%)
Vigilance decline, confusion, disorientation, psychomotor slowing, memory disturbance	19/21 (90%)	3/4 (75%)
Epileptic seizure	3/21 (14%)	0/4 (0%)
Myoclonus	3/21 (14%)	1/4 (25%)
Ataxia/unsteady gait/vertigo	13/21 (62%)	3/4 (75%)
Guillain-Barré-Syndrome (GBS)- like symptoms	1/21 (5%)	0/4 (0%)
Paraesthesia, neurogenic pain	3/21 (14%)	2/4 (50%)
Motor weakness	6/21 (29%)	0/4 (0%)

Please note that the very low case count of VSBV-1 infection does not allow for a direct comparison of both bornavirus encephalitis forms

### Hospital admission, clinical course and outcome

*BoDV-1 Encephalitis.* Following the unspecific, varying disease phase above, all cases at some point showed an abrupt, fulminant neurological progression. The median time between seeking initial medical advice and subsequently being hospitalised with manifest neurological deficits in all patients was two (IQR 0–4) days. Upon admission, half the cases showed typical signs of encephalitis with a history of fever, headache and encephalopathy or focal neurological symptoms. Only three (IQR 2–5) days in median after hospitalisation, cases already required intubation due to rapid neurological and/or respiratory deterioration. During the further clinical course, patients showed a broad range of neurological dysfunction, (focal or generalized) seizures (13/21, 62%), hypothalamic dysfunction (central diabetes insipidus, SIADH, disorder of blood pressure regulation, and/or central fever), and finally developed loss of brainstem reflexes. In the context of encephalopathy at least two cases showed fluctuations in vigilance allowing temporary extubation. In most cases, life-supporting measures were replaced by palliative care due to the poor clinical course and irreversable brain damage, or less often the diagnosis of cerebral death or herniation in the context of a generalized cerebral edema. Two of the 21 cases had prolonged clinical courses of 123 and 293 days between initial hospitalisation and death. Only in one of them, BoDV-1 was diagnosed *intra vitam*, so that this case was treated with antiviral favipiravir. Either case died due to complications two and 10 weeks (the latter under favipiravir treatment) after being discharged in a minimal conscious state.

Death of the 21 BoDV-1 study cases occured a median of 32 (IQR 21–41) days after hospitalisation.

*VSBV-1 Encephalitis.* In all cases, seeking medical attention was followed by hospital admission on the same day. On admission, a triad of fever, headache and neurological signs was not specifically reported. However, fever and any neurological symptom (confusion, myoclonus or paresthesia) were present in three out of four patients and all had at least one flu-like symptom and one sign of neurological involvement. A median of five (IQR 3–14) days after hospitalisation, cases required intubation and mechanical ventilation. During the further clinical course, VSBV-1 infected patients developed unspecific neurological signs and finally coma. One case had a prolonged course of 111 days after hospitalisation allowing extubation and rehabiliation attemps before he died of respiratory failure. In the other cases life-supporting measures were withdrawn due to the poor clinical course and irreversable brain damage. These four patients died 72 (IQR 38–103) days in median after hospitalisation.

**Fig. 2:**
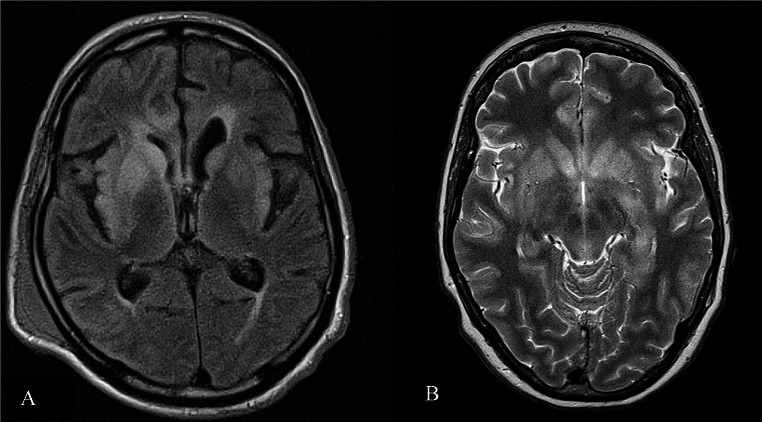
Exemplified MRI images of BoDV-1 and VSBV-1 encephalitis study cases. **A**. Bilateral edema in the basal ganglia and the insula pronounced on the right side in BoDV-1 encephalitis (FLAIR). **B**. Bilateral edema in the basal ganglia and in limbic structures (insula, hippocampus, anterior cingulate) in VSBV-1 encephalitis (T2-weighted)

**Fig. 3:**
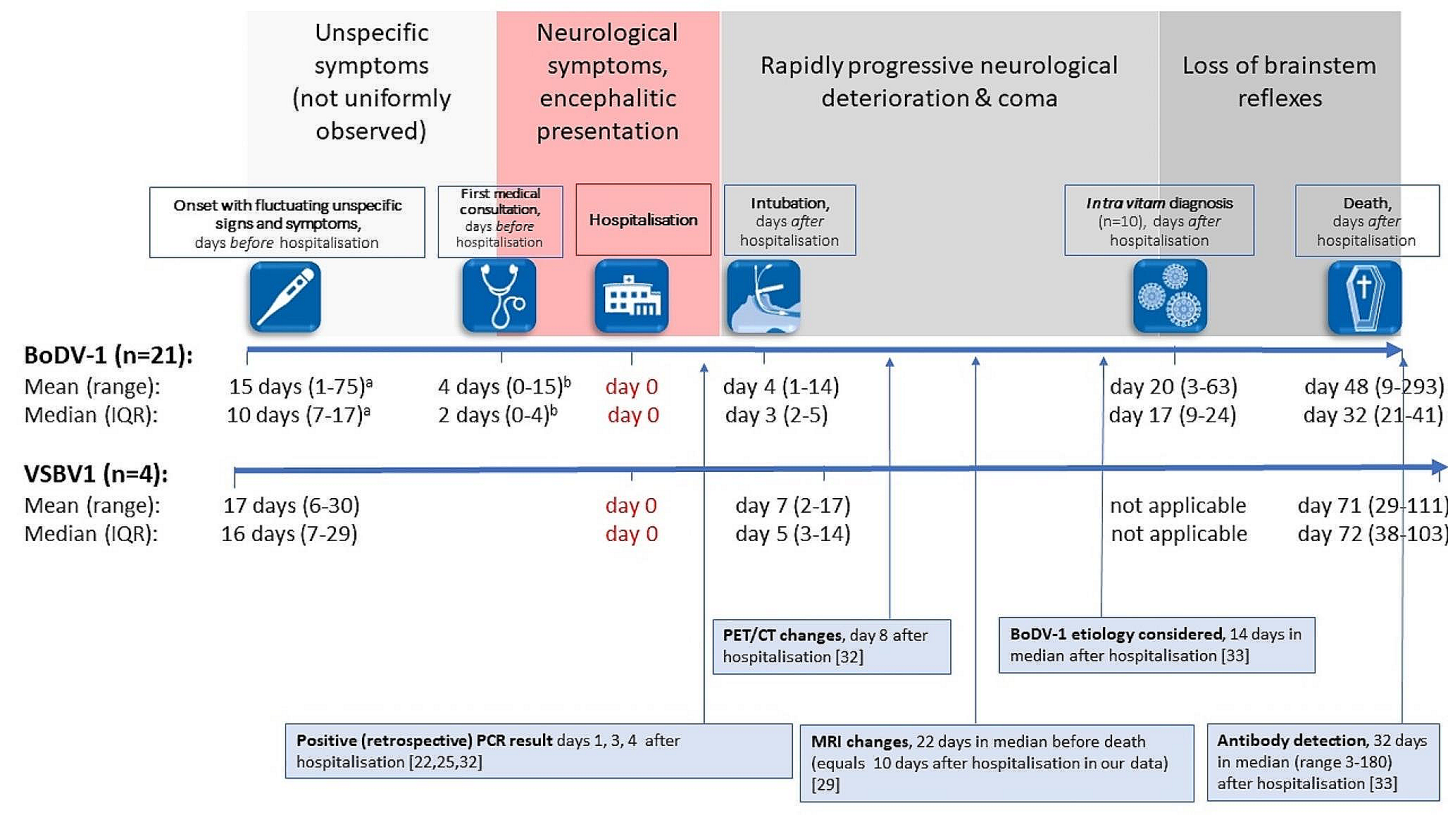
Timeline and clinical course of 21 confirmed acute BoDV-1 and four confirmed acute VSBV-1 encephalitis cases with respect to points in time of specific diagnostic features. Following or occurring during an optional period of unspecific symptoms, such as fever and headaches, patients developed neurological signs, often vigilance decline, leading to hospitalisation. Thereafter, a rapid progression ensued, leading to coma and death after supportive care was ceased in most cases. The phase between the first medical consultation and severe deterioration and intubation (in light red) is extremely short representing the window desirable for diagnosis and treatment start. Please note that the very low case count of VSBV-1 infection does not allow for a direct comparison of both bornavirus encephalitis forms. Also note that the clinical course slightly differed interindividually and that temporary improvement within the course of encephalopathy was observed in individual cases

^a^ data on the exact date of symptom onset is biased due to the insidious onset, *n* = 14 in patients with non-neurological prodromes.

^b^ data on the date of the first medical consultation was available for 18/21 cases.

Table 2 shows characteristics and findings of acute BoDV-1 and VSBV-1 cases in comparison to other viral encephalitis cases. Figure 2 exemplifies cerebral MRI of two study patients. Figure 3 outlines the exact timeline and the clinical course of BoDV-1 and VSBV-1 study patients illustrating points in time of symptom onset, first medical consultation, intubation, diagnosis, and death with respect to published points in time of diagnostic tools.

**Table 2:** Characteristics and findings in BoDV-1 and VSBV-1 encephalitis cases in comparison to encephalitis cases of other viral agents (confirmed and probable) [34]

Characteristics and/or findings	BoDV-1 (*n* = 21)	VSBV-1 (*n* = 4)	Other viral agents (*n* = 170) [34]
Demographics			
Male sex	9 (43%)	3 (75%)	94 (55%)
Age, median years (range)	47 (6–79)	62 (45–72)	33 (0–89)
Clinical			
Days from onset of CNS^a^ symptoms to admission, median (range)	7 (1–75)	8 (2–14)	3 (0–65)
Vigilance decline (confusion, disorientation, memory disturbance, psychomotor slowing) before admission	19 (90%)	3 (75%)	Not specified
ICU^b^ admission	21 (100%)	4 (100%)	81 (55%)
Prodrome or concurrent symptoms			
Fever	19 (90%)	3 (75%)	125 (75%)
Headache	14 (67%)	2 (50%)	Not specified
Respiratory symptom	7 (33%)	3 (75%)	38 (24%)
Gastrointestinal symptom	10 (48%)	2 (50%)	69 (42%)
Rash	0 (0%)	0 (0%)	30 (18%)
Seizure	13 (62%)	0 (0%)	63 (38%)
Coma	21 (100%)	4 (100%)	23 (14%)
Death	21 (100%)	4 (100%)	22 (13%)
Length of hospital stay, median days (range)	31 (9–73)^c^ (*n* = 20)	72 (29–111)	10 (0-1124)

^a^ CNS = central nervous system

^b^ ICU = intensive care unit

^c^ “Length of hospital stay” is not identical with “day of death after hospital admission” as in Fig. 3 since 2/21 patients were discharged from hospital before their death. The date of discharge is unknown in 1/2 patients, therefore *n* = 20 in this variable

### Therapeutic approaches

Since the causative pathogen was still unknown in 11 out of 21 BoDV-1 patients and in all four VSBV-1 patients during hospitalisation, therapy was mostly empiric. Table 3 summarizes therapeutic approaches. Due to the small case numbers and the survival times depending on points in time of treatment cessation, we abstained from a comparison of the different therapy approaches concerning survival times.

**Table 3:** Therapeutic approaches to patients with BoDV-1 and VSBV-1 encephalitis with known therapy (values are to be regarded as minimum values)

Cases with known therapy	Empiric anti- biotics	High dose cortico- steroids	Acyclovir suspecting HSV^a^ encephalitis	Anti- tuberculous treatment	Plasma- pheresis	IVIG^b^	Favi- piravir	Favipiravir with intra vitam diagnosis
BoDV-1 19/21	19/19	19/19	19/19	2/19	8/19	4/19	6/19	6/10
VSBV-1 4/4	4/4	4/4	3/4	1/4	0/4	0/4	0/4	0/0

^a^ HSV = herpes simplex virus

^b^ IVIG = intravenous immunoglobulin therapy

*BoDV-1 Encephalitis.* Experimental therapy with antivirals with in vitro efficacy against BoDV-1 such as favipiravir was administered in 6/10 cases with *intra vitam* diagnosis, either as a monotherapy (*n* = 2), or in combination with ribavirin (*n* = 4). Out of the latter, one received remdesivir in addition to the combination therapy. In 4/6 patients, the exact date of favipiravir initiation is known. In these patients, favipiravir was started a median of 23 days (range 19–66 days) after admission, seven days before death (range 3–21 days). In the other four cases with *intra vitam* diagnosis, treating physicians refrained from experimental antiviral treatment. Supportive immunosuppressive therapy with cyclophosphamide, rituximab, bortezomib, ciclosporin, sirolimus, mycophenolic acid or methotrexate was given in 4/19 cases, partly under the assumption of an autoimmune encephalitis. A significant clinical benefit was only seen in one pediatric case with early antiviral and immunosuppressive treatment [15].

*VSBV-1 Encephalitis.* Ribavirin was administered in one case empirically; none received favipiravir.

## Discussion

Even though laboratory-based, neuropathological and radiological characteristics of bornavirus encephalitis have been studied in detail [16, 22, 25, 27–31, 33, 35] diagnosis of at least incident BoDV-1 cases is still delayed and remains challenging [13, 24, 25, 36], partly due to unawareness among treating physicians [33] and an unknown course of disease [22]. Hence, identifying typical clinical signs and symptoms of bornavirus encephalitis could contribute to an accelerated diagnosis [24] and completes the emerging picture of this disease. However, earlier studies on the clinical presentation of encephalitis caused by different, other viral agents, especially herpes simplex virus (HSV), found no substantial clinical characteristics to be useful diagnostically [37, 38, 44]. According to our findings and demonstrated for the first time, this also seems to be true for bornavirus encephalitis in the early disease phase. In contrast, the abrupt, rapidly progressive vigilance loss leading to coma within three (BoDV-1) and five (VSBV-1) days in median after hospitalisation and seizures in the majority of BoDV-1 patients is strikingly characteristic. This phase of rapid progression is probably the most obvious clinical characteristic of bornavirus encephalitis, besides the incomparably high fatality rate, but also its dilemma: The timeframe for diagnosis (and therefore any therapeutic attempt) is extremely short and requires a high level of clinical awareness.

As in other viral encephalitis cases [34], but in contrast to previous suggestions concerning BoDV-1 [25, 35, 39, 40], our patients with either bornavirus as causative agent did not show a consistent and therefore obvious prodromal phase concerning symptoms or time interval. Without a clear and consistent prodromal phase however, the course of disease becomes even shorter, and thus the possible timeframe for diagnosis and consecutive treatment even smaller. Of interest, noted prodromal (non-neurological) symptoms in our study were heterogeneous, fluctuating, and unspecific and therefore may have obscured diagnosis. At least, however, these prodromal symptoms (e.g. fever, flu-like symptoms) pointed at an underlying infection. Irrespective of optional prodromal signs and a very wide range concerning the time interval of prodromes before hospital admission (1–75 days), on hospital admission all study patients showed severe neurological signs and symptoms. A triad of fever, headache and encephalopathy or focal neurological signs was seen in 52% of BoDV-1 infected cases, clearly hinting towards the diagnosis of encephalitis. This triad was not seen in any of the VSBV-1 infected patients on admission; however, fever and any neurological symptom were present in 3/4. Of note, almost all our study patients were treated for HSV encephalitis, the most common encephalitis worldwide [41], so the treating physicians considered a viral encephalitis at least as a differential diagnosis. Our study adds clinical evidence that patients (in endemic areas or after contact with exotic squirrels) presenting with an initially unspecific (neurological) picture – which rapidly develops into a syndrome consistent with viral encephalitis need to be considered for a specific bornavirus encephalitis work-up, at the best even before fulminant disease progression.

The insidious, fluctuating characteristics of disease onset demonstrated in our systematic analyses for the first time, along with the broad clinical spectrum presumably contributed to delaying not only medical consultation in general, but also diagnosis. Of note, all BoDV-1 cases in this study who received an *intra vitam* diagnosis were already intubated at time of diagnosis and thus in an advanced disease state in an intensive care unit setting - a predictor of a poor outcome in acute viral encephalitis [42]. Diagnosing a bornavirus infection before the development of neurological signs and symptoms seems impossible as demonstrated in our study, especially in the absence of consistent prodromes. However, once neurological symptoms are present, delays in diagnosis may be reducible. In our study, the diagnosis of BoDV-1 was established as late as day 17 after hospitalisation in median with patients being already critically ill. In line, a recent study showed that the etiology of BoDV-1 was considered for the first time on day 14 after hospitalisation in median [33]. Whereas MRI changes and seroconversion appear late in the disease course [25, 29, 33], (retrospective) PCR in CSF was shown to be positive shortly after hospital admission [15, 22, 25, 32], but has a poor negative predictive value in general [7]. The variable presence of bornavirus-reactive antibodies in serum and CSF (at least for BoDV-1) [5, 7, 22, 33] also requires repeated testing. Thus, even if patients presented or were tested earlier due to raised awareness (preferably within the critical light red phase shown in Fig. 3), initial virological laboratory testing might still be negative. Fortunately, diagnosis has been achieved faster in recent years [33], at least in regions where BoDV-1 is known to be endemic.

CSF changes can be mild or even absent in both BoDV-1 and VSBV-1 encephalitis, and they are similar to other viral encephalitis changes encompassing mild lymphocytic pleocytosis [27, 28]. In contrast, MRI abnormalities are characteristic (as exemplified in two of our study patients) with symmetric T2-hyperintensities of the caudate nucleus and the limbic system including the insula, however occurring late [16, 29], and mimicking Creutzfeldt-Jakob disease (CJD), an important radiological differential [29], besides autoimmune limbic encephalitis showing affection of the limbic system with abnormalities on T2-weighted MRI sequences [43]. Both radiological differential diagnoses however differ from bornavirus encephalitis clinically, epidemiologically and in the laboratory work-up.

A recent case study showed 18-fluorodeoxyglucose PET/CT as a possible early diagnostic tool clearly preceding MRI changes [32], but using PET/CT for bornavirus diagnosis still requires clinical awareness and remains a sophisticated, cost-intensive imaging technique not generally available or used for primary diagnosis. Speeding up diagnosis with clinical awareness and new diagnostic tools seems desirable on one hand, but obviously remains a challenge. On the other hand, without an established therapy the outcome can hardly be improved.

Empiric antiviral treatment as recommended in HSV encephalitis to hit early in the disease course while awaiting diagnosis sounds desirable but far away for patients with suspected bornavirus encephalitis. Until now, not even a treatment once the diagnosis is confirmed is established, and data for virostatic favipiravir are limited to in vitro experiments. In our patients, significant and lasting clinical improvement under antiviral therapy with in vitro efficacy, either in monotherapy or in combination, could not be seen, possibly due to critically delayed administration as demonstrated in our study for the first time: Targeted antiviral therapy was only started seven days before death in median as an individual salvage attempt, shortly after the diagnosis was confirmed. Therapeutical dosages, pharmacokinetics, and interactions of the most promising (oral) favipiravir are not known for bornavirus encephalitis. Whether or not these antivirals have a significant effect when administered earlier, dosed correctly, in combination with intensified immunosuppression [15, 30], and supported by therapeutic drug monitoring, needs to be analyzed. In a recent pediatric case report [15], the fatal outcome despite early intensive antiviral and immunosuppressive therapy with initial significant improvement probably involves a number of causes, but ultimately remains unexplained. Also, survival over years (but with severe neurological sequelae and in need of long-term care) independent of initial treatment attempts and comorbidities has been reported in singular cases of both bornavirus encephalitis forms and remain similarly ambiguous [2, 5, 23]. On a side note, empiric immunosuppressive drugs had been given in the cases described here, and likely prolonged survival.

The majority of the bornavirus cases in this study died after treatment cessation on the presumed basis of an overwhelmingly poor neurological outcome, irreversible brain damage, and the lack of an effective targeted therapy. This is probably true for the vast majority of all known cases. Survival times are therefore hard to interpret and the wide range concerning the time of death in our study (9-293 days after hospitalisation for BoDV-1 and 29–111 for VSBV-1) are to some extent arbitrary. The tendency of VSBV-1 infections to have longer disease durations than BoDV-1 as seen in our study and as discussed before [5], might therefore be an artefact.

Of note, a discrimination between the two bornavirus encephalitis forms is possible neither by MRI [16], nor by histopathology [28, 31], owing to highly similar imaging features and tissue destruction characteristics. Our investigation adds that the clinical picture also does not allow for a discrimination (for academic and public health reasons), even if the low VSBV-1 case count hinders direct comparison. Besides epidemiological considerations, discrimination can be achieved by virus-specific serology, PCR and sequencing [5], or in situ-hybridization in brain tissue [5, 31].

This dual case series provides the broadest comprehensive and comparative clinical overview of the two etiologic agents of bornavirus encephalitis available to date. We were able to provide data on a substantial proportion of all bornavirus cases documented so far. The data for the onset of symptoms and possible prodromes for BoDV-1 is not only based on medical records, but on medical interviews. However, we cannot entirely rule out that the results may not be generalizable to all cases. Our investigation demonstrates that insidious onset, rapid progression and extreme fatality are the clinical characteristics of bornavirus encephalitis resulting in a very small timeframe for repeated diagnostic procedures encompassing neuroimaging, serology and CSF analysis. Our results add substantial evidence and complete the knowledge about the clinical picture of bornavirus encephalitis. However, reducing the fatality rate of bornavirus encephalitis in the near future by timely diagnosis and targeted therapy remains not only challenging but overall questionable to be reached. Studies not only on the prevention but on diagnostic accuracy, possible surrogate markers, as well as on the therapy of human bornavirus encephalitis and guidelines for the (causal or palliative) treatment of future *intra vitam* diagnosed cases are urgently needed to reduce both, morbidity and mortality, of this severe disease.

## Data Availability

Data not available due to privacy/ethical restrictions.
